# Promoting human behaviour change in a first opinion veterinary clinic to improve home oral hygiene in pets utilising the 5A framework

**DOI:** 10.1371/journal.pone.0362316

**Published:** 2026-11-13

**Authors:** Lowri Elis-Williams, Fiona McDowall, Claire Harrison, Kirsty Hughes, Arlene Crawford, Jill R. D. MacKay

**Affiliations:** 1 Hospital for Small Animals, Royal (Dick) School of Veterinary Studies, University of Edinburgh, Easter Bush, Midlothian, Scotland, United Kingdom; 2 Veterinary Medical Education Division, Royal (Dick) School of Veterinary Studies, University of Edinburgh, Easter Bush, Midlothian, Scotland, United Kingdom; 3 Animal Welfare Centre, Royal (Dick) School of Veterinary Studies, University of Edinburgh, Easter Bush, Midlothian, Scotland, United Kingdom; Maulana Abul Kalam Azad University of Technology West Bengal, INDIA

## Abstract

The welfare of companion animals is predicated by caregiver management and decision making, and so the veterinary consult is a key intervention point to improve animal welfare. As with many health-related behaviours, instigating long-term changes is a challenge in the veterinary profession, with knowledge-deficit models of human behaviour change proving ineffective at promoting positive welfare for companion animals. Dental care is one of the most prevalent health disorders in the UK, with many pets receiving suboptimal Home Oral Hygiene (HOH) care. This study implemented a Dental Care clinic in the Royal (Dick) School of Veterinary Studies Small Animal General Practice and recruited 61 caregivers to a retention-based study looking at changes to HOH. The Dental Clinic followed the 5As framework, adapted for veterinary use, to promote clinically relevant HOH behavioural changes. Caregivers were surveyed prior to the clinic consult, after the final follow-up consult, and at a six month retention date to explore their HOH. Retention was a challenge in the project, with only 39 caregivers attending the clinic, 21 (54%) responding to the End of Clinic survey and 13 (33%) responding to the six month post survey. HOH methods were rated on a 9 point scale, and in the End of Clinic Survey, HOH score had improved by 2.62 points (t _student_ = 2.77, *p* = .01, g _Hedges_ = 0.58, 95% CI[0.13,1.02]), however, the study was underpowered to detect significance at the Retention stage (F_Fisher_(1.64, 19.63) = 3.28, *p* = .07, ω^2 _p_ = 0.08, 95%CI[0.00, 1.00]). We discuss the applicability of the 5As framework in veterinary behaviour change, and implications for practice.

## Introduction

Companion animals, as an animal population, are heavily reliant on their caregivers to make informed decisions regarding their health and wellbeing [1]. It can be difficult to get direct indications of the companion animal population size, but companion animals represent a large and relatively long-lived animal population. The UK companion dog population was estimated to be between 8.54–15.16 million animals in 2019 [2] with the average UK dog in that period living between 11.19–11.27 years [3], whereas there were an estimated 10.8 million cats in the UK households in 2021 [4], with cats considered ‘mature’ at 7–10 years and geriatric from 15 years [5]. Since 2010, more US households have a dog than a child [6], with many caregivers internationally considering companion animals to be part of the family, and indeed, childlike to them [7,8]. Companion animal management is therefore as varied as individual families can be, in contrast to other large managed animal populations such as production animals, which are often regulated and standardized by food safety legislation and welfare standards [9].

Health is a fundamental component of animal welfare, influencing affective state and behaviour [10], and has an important impact on the wellbeing of companion animals. Periodontal disease is a progressive condition which has significant impact on the welfare of the animal [11–13], and affect a range of companion animals from dogs [14] to cats [15] and rabbits [16]. Periodontal disease is one of the most common conditions affecting cats and dogs in the UK [14]. It has a complex pathology which is initiated by oral bacteria found in the plaque matrix which accumulates on the crown of the tooth (supragingival). The plaque extends under the gingival sulcus (subgingival) where the bacteria cause inflammation of the gingiva (gingivitis) and leads to periodontitis (inflammation and destruction of the periodontium, the supporting structures around the tooth). Periodontitis results in pain, infection, alveolar bone loss and eventual tooth loss if left untreated [13]. Periodontitis is irreversible once established with treatment options including periodontal surgery to promote reattachment of the periodontium or, more commonly, extraction of affected teeth. As such, removing plaque is an extremely important preventative and therapeutic treatment for early periodontal disease (gingivitis). There are a range of methods that caregivers can use to achieve this, and support their companion animal’s periodontal health, including manual removal through brushing and certain food or chews, and the provision of active ingredients in paste, food, or water [17]. The methods available vary in effectiveness of plaque removal, time commitment, daily financial cost, and ease of use providing us with a Spectrum of Care options which the clinician can advise the most suitable for the client and patient [18]. Despite this relatively simple recommendation, periodontal disease remains highly prevalent in companion animal populations [14,19,20], suggesting that Home Oral Hygiene (HOH) management remains suboptimal in these populations. Plaque will build up within 24 hours of brushing, therefore HOH must be performed daily [13].

A reasonable ‘goal’ for companion animal welfare would be to support caregivers to achieve better HOH practice, thus reducing periodontal disease in the companion animal population. Indeed, research has suggested that raising awareness of breed impacts on periodontal disease might improve welfare [14]. Despite an increasing evidence base for good welfare in companion animals, this high quality evidence has not translated to better management practices [21,22]. The theory that caregivers are simply unaware of what needs to be done is known as the Knowledge Deficit Model in human behaviour change, with the assumption that a change in knowledge will promote a change in attitude which will change practice [23]. This approach has been demonstrated as largely ineffective in animal welfare [24,25], and reflects an ongoing lack of underpinning human behavioural change theory in the promotion of animal welfare interventions [26]. To support animal welfare, interventions must incorporate human behavioural change theories which recognise the complexity and uniqueness of animal management, particularly in companion animal management [27,28]. Any human behaviour change approach used in companion care must be adapted for the unique situation where we have both a patient and a caregiver. Approaches must take into account all aspect of that patient, caregiver, and the wider context to ensure that a care plan is appropriate for those individuals [29]

Much of the current work around supporting animal welfare through human behavioural change has been limited in applying human behavioural change theory [27]. A recent review in production animals [26] found that most human behavioural change studies focus around Theory of Planned Behaviour Change, and most studies focused on behavioural intention, rather than the behaviour itself. These studies were also not related to clinical care behaviours, e.g., behaviours that we would expect to see as a consequence of clinical consultation. One human behavioural change approach which has seen limited veterinary application, but has been shown effective in a range of human clinical settings is the 5As framework [27]. The 5As framework is not a model intended for research, but was developed in tobacco addiction treatment as a clinical approach to changing behaviours [30]. The framework inherently reflects the uniqueness of each individual patient and client context and ensures that individual needs and barriers are centred in any treatment plan [31,32]. While the framework was initially developed to promote the cessation of a behaviour (smoking tobacco) it has been used in settings to promote other health conscious behaviours, such as healthy practices for weight loss [33]. It has also been utilised in paediatric settings for weight loss incorporating caregiver interactions with patient behaviours [34,35]. We were interested in whether the 5As framework could support animal caregivers to promote HOH behaviours for two key reasons. While many other behavioural change models have been utilised in animal welfare contexts, such as Theory of Planned Behavioural Change, Behavioural Change Wheel, the Health Action Process Approach, and COM-B models, their use is centred around designing an educational intervention, and occasionally testing it [26]. Clinical treatments do not always benefit from conceptualised as educational interventions, and this impacts how behavioural change theories can be utilised within them [36]. The 5As framework was specifically designed and utilised for use in a clinical setting, and has been found effective in this setting. It required minimal training for veterinary clinicians to use, and could be quickly integrated into clinical practice. Additionally, as the 5As framework had been successfully utilised in paediatric settings, we were interested in whether it was flexible enough to adapt to the veterinary triad of care between practitioner, animal caregiver, and animal. In this study, we explored the utility of the 5As framework in veterinary practice with a view to supporting HOH practices. We instituted a Dental Clinic with a consult plan based on the 5As framework, and recruited participants to evaluate long term behavioural changes related to HOH following the clinic consults. The 5As framework has clearly set out actions for the clinician (Ask, Advise, Assess, Assist, Arrange) which can be easily incorporated into routine General Practice utilising vet and nurse core communication skills, unlike other human behavioural change approaches such as motivational interviewing and the COM-B model which require personnel training and advanced, new, or additional communication skills. To our knowledge, this is the first research study exploring the 5As framework in the veterinary context.

We hypothesised that:

H^1^_0_: Caregiver knowledge regarding Home Oral Hygiene method effectiveness would not change following the dental clinic
H^2^_0_: The caregiver self-reported frequency of effective Home Oral Hygiene behaviours would not change following the dental clinic
H^3^_0_: Overall caregiver Home Oral Hygiene behaviour would not improve following the dental clinic.

## Methods

### Ethics and availability

This project was granted ethical approval by the Royal (Dick) School of Veterinary Studies Human Ethics Review Committee (Reference: HERC_2024_005).

Data and code for analyses are hosted on the Open Science Framework (https://osf.io/x5mdy)

### Researcher position statement

Ideological biases permeate and influence researcher behaviour even when researchers profess highly positivist and empirical theoretical stances [37], so we follow 2025 issued recommendations [38] to consider what personal experiences and characteristics are likely to impact the presentation and analysis of data. JRDM was the lead on study design and analysis, and is not a veterinary professional, but has worked in animal and childcare settings for a number of years. She describes herself as principally a methodologist working in veterinary education and animal welfare, with a socio-constructivist epistemological position. LEW is general practice veterinary surgeon with an academic interest and teaching roles in Veterinary Communication client experience, and contextualised care. She is developing her research perspective and is largely post-positivist, reflecting her clinical veterinary training. CH is a registered veterinary nurse with a Veterinary Technician Specialty in Dentistry and a background in clinical veterinary nursing and veterinary dentistry education, FM is a registered veterinary nurse with an oncology speciality and a lecturer in veterinary education with a background in psychology and communication, and AC is a veterinary receptionist. FM, CH and AC did not contribute to the data analysis, and so epistemological positions are not relevant. KH is a veterinarian and researcher in veterinary education, with a socio-constructivist epistemological position. Additionally, we have made a number of conscious decisions in our language, referring to animal caregivers due to ongoing concerns with the ethics of business in veterinary care [39,40], and referring to animals as he/she/they instead of ‘it’ [41].

### Study design

This study was divided into four distinct phases: Recruitment, Initial Consult, Follow-Up Consult(s), and Retention. We utilised the 5As Framework of Behavioural Change [30] within the Dental Clinic to identify and promote enduring behavioural change with relation to Home Oral Hygiene behaviours. A series of three surveys, in the Recruitment, End of Clinic and Retention phases assessed the caregiver’s knowledge, HOH behaviour frequency, and satisfaction with the clinic.

### Adapting the 5As framework for veterinary use

The 5As strategy for treating tobacco use and dependence [30] was minimally modified to allow for the change in use in promoting effective HOH behaviours (Table 1). Within the ‘Assist’ step, a contextualised care approach [18] was utilised to evaluate each caregiver-patient dyad’s scope for change. We highlight to readers that we provided our recommendations with a stated optimum care plan, e.g., mechanically brushing teeth with a paste, following clinical recommendations [13], but that for each caregiver-patient dyad there was a individualised care plan set out which may have recommended a less-optimal approach per clinical recommendations, but was contextualised to that dyad’s requirements. There were additionally some minor changes to structure, e.g., the recommendation in the 5 A’s method to provide social support could not be fulfilled because there are fewer resources to support pet owners with HOH than resources for ceasing tobacco use (in humans). Existing resources were used [42,43] and a separate resource document (see supplementary material Appendix A) was created by the authors.

**Table 1:** Modifications to Fiore et al (2000) 5As framework for dental veterinary clinic.

5A steps	Fiore et al	Veterinary Dental Clinic	Reasoning for change/notes
Ask	Implement an office-wide system that ensures that, for every patient at every clinic visit, tobacco-use status is queried and documented.	Ask what HOH is being used daily at clinic	There was no scope for a practice wide change in this study. *Note – assessing the patient’s oral health is done as standard at every annual health check*
Advise	In a clear, strong, and personalized manner, urge every tobacco user to quit.	Gave clear, strong, personalised recommendation to start/improve Oral Hygiene. Include examples and advice will be tailored to patient and family	Change from cessation of behaviour to initiating a new behaviour
Assess	Ask every tobacco user if he/she is willing to make a quit attempt at this time (e.g., within the next 30 days).	Ask if caregiver willing to start/increase Home Oral Hygiene?	Change from cessation of behaviour to starting a new behaviour
Assist	Help the patient with a quit plan. Provide practical counselling (problem solving/skills training) Provide intratreatment social Support. Help patient obtain extratreatment social support Recommend the use of approved pharmacotherapy except in special circumstances. Provide supplementary materials.	Agree on patient and caregiver centred plan with the caregiver Provide a demonstration of how to use products. Discuss potential challenges and offer solutions No social support was offered Provide print out/email dental advice handout. Highlight relevant aspects. Include product recommendations	Contextualised care for patient and caregiver, rather than patient centred due to veterinary setting The authors are not aware of any current social support and creating social support network is beyond the scope of this study. A caregiver information sheet out was created by the authors explaining each HOH method and advice on using it. (Appendix A) A list of VOHC approved products available in the UK was included A link to the WSAVA tooth brushing guide was included in the post-consultation email
Arrange	Schedule follow-up contact, either in person or via telephone.	Book follow-up phone call for 4–8 weeks	Interval to be agreed with the caregiver and clinician

A consult template was created based on the modified 5As framework. The consult template allowed for consistency between clinicians and acted as a reminder to ensure all steps were fulfilled.

Prior to discussing the modified 5As with the caregiver, a clinical assessment was made of the patient. The clinical assessment was designed to identify appropriate HOH methods for each patient. Presence of mouth pain and the patient’s temperament were assessed to ensure advice given was clinically appropriate for that individual patient, taking into account the patient’s behavioural needs, personality, the caregiver’s finances, capacity, and physical capabilities to recommend advice that was achievable for the caregiver-patient dyad, and of clinical benefit. This meant, for example, that some caregivers were not recommended mechanical toothbrushing if the patient would not tolerate the procedure. During the Arrange step, at follow-up, the personalised plan was re-assessed to explore whether the individual care plan was working, and changes could be made if not, with further follow-up possible.

### Recruitment phase

In the Recruitment Stage, caregivers attending the Dick Vet General Practice (DVGP) were verbally invited to the study and asked for consent to receive an email invitation. Any caregiver attending in the period from March 2024-September 2024 for any cat or dog vaccination appointment were eligible for invite, which amounted to approximately 530 caregivers based on average vaccination consult frequency. Within the next week, consenting caregivers received an email with a link to our Recruitment Survey, which collected data on their demographics, their pet’s details, and their current understanding and perceptions of their pet’s dental health (see Survey Design below). Weekly, these responses were collated by JM who forwarded contact details only to DVGP staff to arrange the Initial Consult with the caregiver. We aimed to recruit 50 caregivers to the survey, which would represent approximately 10% of caregivers seen in that period.

### Dental clinic initial consult

The Initial Consult involved an appointment with the Dental Clinic, where a clinical exam was conducted, a clinically relevant history taken, and then a clinical assessment of which Home Oral Hygiene methods were acceptable in this patient. Each practitioner was free to make their own assessment on acceptable methods for example ensuring that any HOH method chose would not worsen any mouth pain, or not recommending brushing if the patient’s temperament could put the client at risk of being bitten. The consult template (see supplementary information) was followed during each consultation and all consults were run by LEW, a general practice veterinarian, or CH, a veterinary nurse (VTS dentistry). The 5As were incorporated into the consultation as follows: The caregiver was first **asked** which Home Oral Hygiene Methods were being used daily. The caregiver was given personalised **advice,** with examples tailored to the patient and lifestyle. The clinician made an **assessment** as to whether the caregiver was willing to start or adapt their Home Oral Hygiene approach. The clinician then **assisted** the caregiver to agree on a patient and client centred plan, with a demonstration, discussing the potential challenges with solutions, and giving the caregiver supplementary materials highlighting information relevant to the caregivers personal circumstances. Finally a follow-up call was booked for 4–8 weeks to **arrange** a further check. A follow up phone appointment was booked at the end of each consultation with confirmation sent via email along with a consultation summary as per the template guidelines.

### End of clinic phase

The follow-up call followed a similar format, with caregivers being consulted on what changes they had made, and then a further follow-up arranged if the 5A protocol suggested it would be useful. When caregiver and practitioner had achieved a mutual end, a follow-up email was sent including a summary of the plans made and a link to the End of Clinic Survey.

### Retention phase

Six months after the caregivers last follow up call, a Retention Survey was sent via email. Up to two reminder emails were sent after each of these surveys if there was no response within 2 weeks. If there was no further response, the caregiver was considered to have exited the study.

### Survey design

There were three surveys in this project, the Recruitment Survey, the End of Clinic Survey, and the Retention Survey. The Recruitment Survey contained questions relating to the caregiver and pet demographics, and assigned a unique identifier which the caregiver would be able to use to respond to the following two surveys. It asked questions about the pet’s dental health and history, and the caregiver’s hopes regarding dental health. The End of Clinic Survey asked about the caregiver’s experiences in the clinic, their satisfaction, and a series of questions aimed at checking whether the 5As protocol was followed in the consult. Finally, the Retention Survey asked caregivers about their perceptions of their pets’ overall dental health and any changes since the start of the project, and whether caregivers felt they had achieved their goals. The Recruitment and the End of Clinic Survey contained a knowledge question asking participants to rank six HOH methods in order of effectiveness (Dental supplements to food or water, dental chews, diets tailored specifically for dental health, gel or paste added as a supplement, mechanical brushing (with brush or silicone tool) alone, mechanical brushing (with brush or silicone tool) with a gel or paste). All three surveys contained a behavioural frequency question, asking caregivers how frequently they performed the six HOH methods on a scale from: I never use this, less often than weekly, at least weekly, at least five times a week, at least once a day.

The surveys were piloted with 10 friends, family and colleagues and minor changes to question wording were made.

### Scoring home oral hygiene methods

As HOH methods can be varied in mechanism, active ingredient, and frequency, we devised a scoring system to indicate clinically effective changes to HOH practice, based on recent guidelines reviews [11,13] and reviewed by LEW and CH. For example, a caregiver may change behaviour from rarely wiping their pet’s teeth with a wipe to wiping their pet’s teeth once a week, but while this is an increase in frequency, it is not clinically relevant and would not positively impact dental health. Therefore, the scoring system considers mechanism, active ingredient, and frequency, to provide each caregiver-animal pair with a HOH score. A decrease in score reflects positive clinically relevant changes to HOH behaviours, whereas an increase in score reflects negative clinically relevant changes. The scoring system was available for reference by the clinician and caregiver during the in-person consultation and is detailed in Table 2. The HOH Grading scale demonstrates the clinical effectiveness of each daily HOH method. While the HOH grading scale was primarily used as an outcome measure to determine whether clinically-relevant improvement in HOH practice was observed, it was also, used as a guide by the clinicians and clients to pick the best HOH for each individual patient and client with shared decision making [44].

**Table 2:** Home oral hygiene methods scoring system.

Score	Home oral hygiene grading scale
1	Brushing at least once a day AND food AND/OR chews at least once a day (+/- gels/sprays, food/water additive)
2	Brushing at least once a day AND gel/sprays AND/OR food/water additive at least once a day
3	Brushing at least once a day
4	Food AND/OR Chews at least once a day AND gel/sprays AND/OR food/water additive at least once a day
5	Food at least once a day (+/- Chews)
6	Gel/sprays at least once a day AND food/water additive at least once a day
7	Gel/sprays at least once a day OR Chews at least once a day
8	Food/water additive at least once a day
9	Nothing (or any of above fewer than once a day)

### Qualitative clinic reflection

We were also interested in the experience of the practitioners following the clinic’s conclusion. A researcher previously unattached to the project (KH) was brought on to run semi-structured interviews with LEW and CH. These ran in February 2025 after the dental clinic had concluded. The interview schedule is available in the data repository. Interviews were run via Microsoft Teams and transcribed by that program. KH checked transcriptions for legibility.

### Data analysis

To address H^1^ (Caregiver knowledge regarding Home Oral Hygiene method effectiveness would not change following the dental clinic) we asked caregivers in the Recruitment and End of Clinic phase to rank six methods of Home Oral Hygiene in terms of what they considered most effective. We used ‘Score Voting’ [45] to rank the six methods where non-scores were considered as being non-preferred. This approach is similar to committee rankings of grant proposals [45] and allows for a stated ranking as opposed to summing only the medians. We then characterized difference in knowledge across the Recruitment and End of Clinic time points.

To address H^2^ (The caregiver self-reported frequency of effective Home Oral Hygiene behaviours would not change following the dental clinic), we used Welch’s ANOVA to compare between time points [46].

To address H^3^, we scored each caregiver’s HOH as described, and then explored differences in HOH score between Retention and Post Consult stage (n = 21) via Student’s T-Test, and across all three stages (n = 13) via ANOVA.

All statistical analyses were performed in R [47], and utilised the tidyverse [48], likert [49], vote [45], patchwork [50], nord [51], and ggstatsplot [52] packages.

We also utilised reflexive thematic analysis to explore the two semi-structured interviews. JM conducted the analysis and it was sense-checked by KH. Reflexive thematic analysis was considered appropriate for this approach due to its inbuilt acknowledgement of the need for reflexivity, understanding the context of the researcher, and the perspectives brought by the person analysing the data [53,54], and due to its flexibility in being able to be utilised for smaller qualitative datasets such as this one [55,56].

## Results

### Participants and retention

In total, 105 caregivers gave consent to be contacted. Of these, 61 (58%) responded to the Recruitment Survey. One caregiver responded for two dogs. While caregivers were encouraged to attend the clinic with as many animals as they wanted, they were asked to answer the research survey regarding only one animal. This caregiver only responded to the Recruitment Survey, so we arbitrarily selected the first animal they provided data about and disregarded the second, resulting in 61 caregiver-animal pairs. Of the 61 pairs, 39 had an in-person appointment at the clinic (64%). There were 22 caregiver-animals pairs who responded to the Recruitment Survey but did not receive an appointment. For 4 (18%), the available times were not suitable, and for 3 (14%) the trial ran out of appointment times before they were able to attend. For the remaining 15 (68%) we were unable to collect data on why they did not attend the clinic.

21 (36%) responded to the End of Clinic Survey and 15 (24%) responded to the Retention survey. Retention in clinical trials is a common challenge [57], and while there are few meta-analyses of retention in veterinary trials, in paediatric trials, retention can run from 68%−92% [58], so 24% can be considered low, and potential reasons for this are discussed below. Due to a member of staff being on parental leave, there was a change to how participants were reminded of the Retention Survey, resulting in some participants responding to the Retention Survey without responding to the End of clinic Survey, meaning there are only 13 participants (21%) who were followed throughout the project.

### Participants

Caregivers were generally the principle caretaker or joint principle caretaker of their pet and spent a large proportion of time with their pet. 87% of respondents brought a dog to the clinic, and 13% brought a cat (Table 3).

**Table 3:** Caregiver and pet demographic data for 61 respondents.

Question	Responses	n	Percentage
Pet Type	A dog	53	87%
	A cat	8	13%
			
Pet Sex	Male	31	51%
	Female	30	49%
Castration Status	Neutered (castrated)	25	41%
	Not neutered/intact	6	10%
	NA (e.g., female)	30	49%
Spay Status	Neutered (spayed)	25	41%
	Not neutered/still has seasons	5	8%
	NA (e.g., male)	31	51%
Pets Primary Caretaker	I am my pet’s primary caretaker	39	64%
	It is an even split between me and other household members/other households	22	36%
Pets Living Situation	Mostly/always in the house	52	85%
Pets Living Situation	Mostly/always in the house but can access a garden without needing a door opened for them (e.g., has a doggy door)	8	13%
	Mostly/always outside the house or in a garden/kennel	1	2%
Feeding Responsibility	Mostly / always me	36	59%
	Evenly split between me and other members of my household	24	39%
	Mostly / always other members of my household	1	2%
Dental History	Yes, my pet has had some teeth removed by a veterinary professional	10	16%
	No, as far as I am aware, my pet has all their teeth.	50	82%
	Yes, my pet has some teeth missing, but I do not know the cause (e.g., they were missing before you adopted/obtained your pet)	1	2%
Has your pet ever had teeth removed?	Not sure	9	15%
	No	27	44%
	Yes	25	41%
How many hours did you spend with your pet in the average day last week?	mean	14.14	
	median	12.50	
	min	4.00	
	max	24.0	

The majority of animals (82%) had all of their teeth, so far as owners were aware, and 63% of caregivers reported that their pet occasionally or more often had an unpleasant breath odour. While most (70%) caregivers felt they had a little or good knowledge regarding dental health, 79% of caregivers wanted to be managing their pet’s dental health differently (Fig 1).

**Fig 1:**
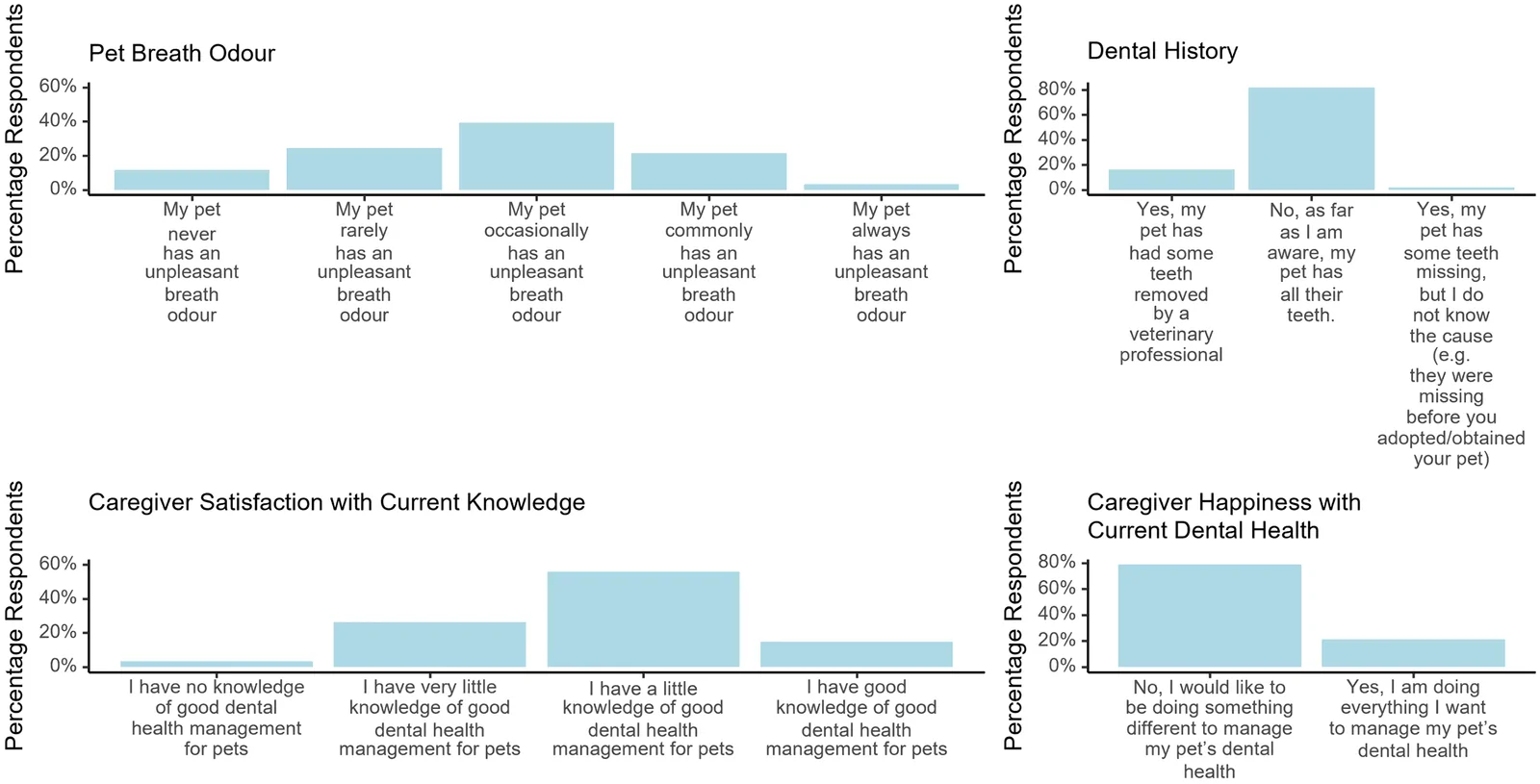
Current dog dental symptoms, history, and caregiver happiness with dental health and knowledge (n = 61).

### Changes to knowledge

In the Recruitment phase, in Condorcet voting run off, Mechanical Brush with a Paste or Gel and Mechanical brushing were rated 6 (Most Effective) and 5 respectively, with Gel or Paste alone and Supplements being considered a 2 and 1 (Least Effective). This suggests that pre-clinic, knowledge was generally good, at least in terms of identifying brushing as the most effective solution. In the Post Consult period, Mechanical Brush with Paste or Gel remained the most effective solution, per Condorcet voting run off, but Chews overtook the Mechanical Brush (Fig 2)

**Fig 2:**
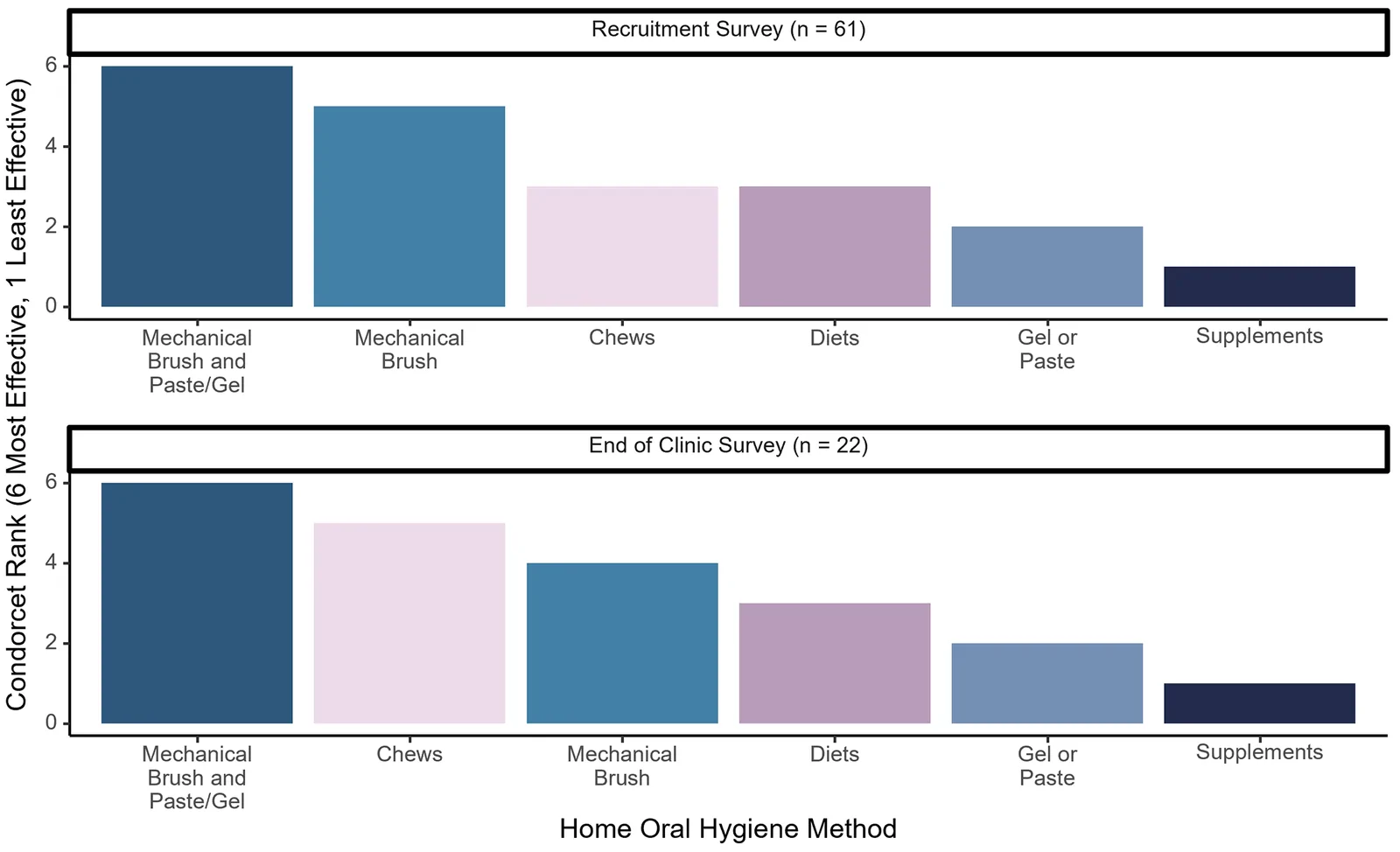
Results of Condorcet Voting Run-Off when caregivers were asked to rank the effectiveness of Home Oral Hygiene Practices at Recruitment (n = 61) and Post Consult (n = 22).

### Changes to behaviour

Participants were asked how frequently they conducted each HOH method at all three survey points, and while the Retention behaviours were not significantly different from Recruitment Behaviours, Chews were provided more frequently (F _Welch_ (2,33.95) = 4.13, ω ^2^_p_ = 0.14 CI _95%_ [0.00, 1.00], *p* = .02) at both End of Clinic and Retention than they were at Recruitment, and Mechanical Brush and Paste was performed more frequently at End of Clinic in comparison to Retention (F _Welch_ (2,27.53) = 10.51, ω ^2^_p_ = 0.39 CI _95%_ [0.13, 1.00], *p* < .001), but these behaviours were only significant between Recruitment and End of Clinic, not between Recruitment and Retention (*p*_Holm-adj._ < .001, Fig 3).

**Fig 3:**
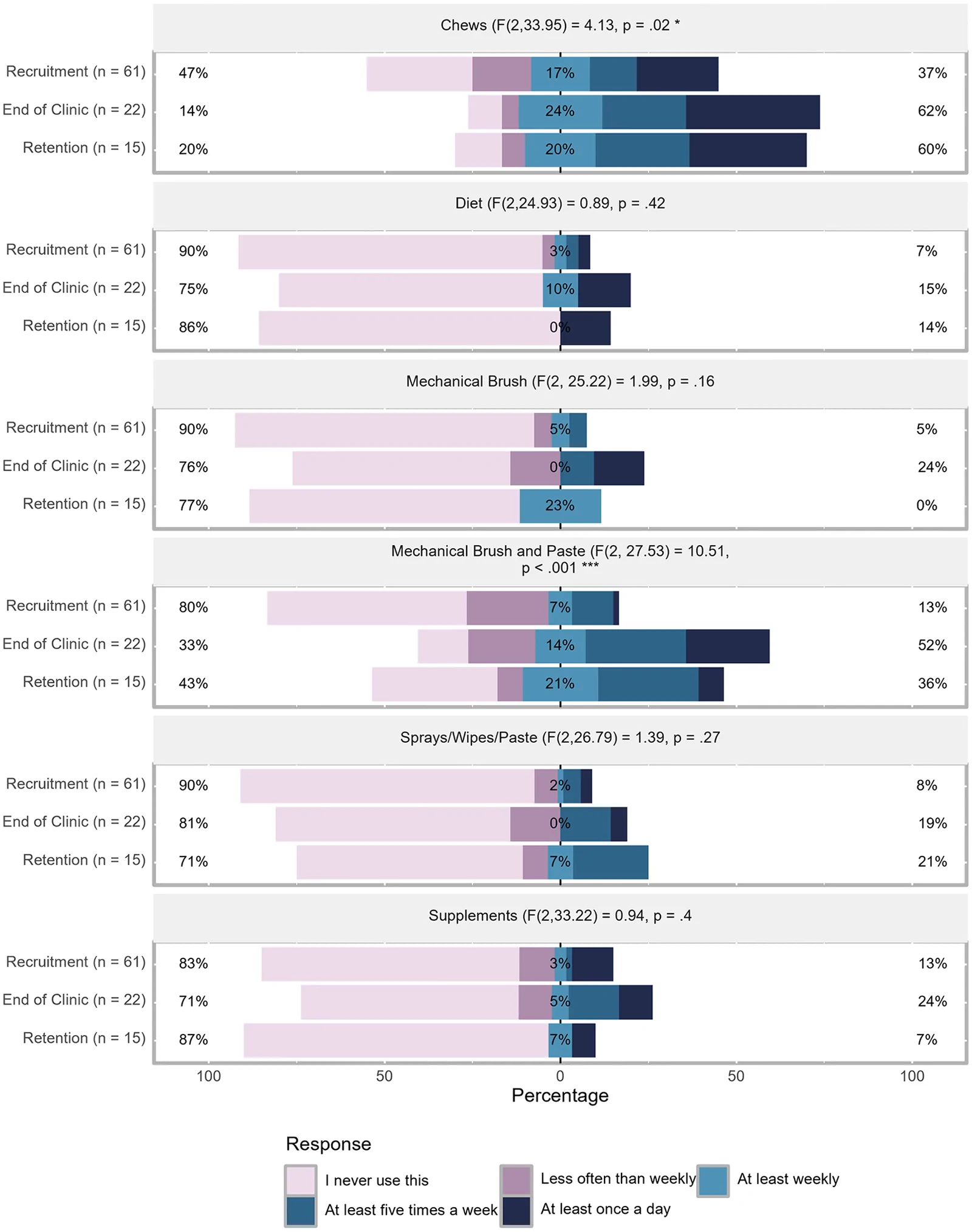
Differences between home oral hygiene behaviour frequencies between recruitment, end of clinic, and retention surveys compared by Welch’s ANOVA.

### Changes to home oral hygiene score

Across all three survey points, the Recruitment Survey had the highest HOH score (i.e., least clinically effective management), and the End of Clinic phase had the lowest (i.e.,. most clinically effective management). Looking at only the 21 participants who engaged with the clinic and responded to the End of Clinic survey, we see a significant improvement in clinically relevant HOH practice (Fig 4), with average score decreasing from 6.48 to 3.86, a moderate effect size (t _student_ = 2.77, *p* = .01, g _Hedges_ = 0.58, 95% CI[0.13,1.02]).

**Fig 4:**
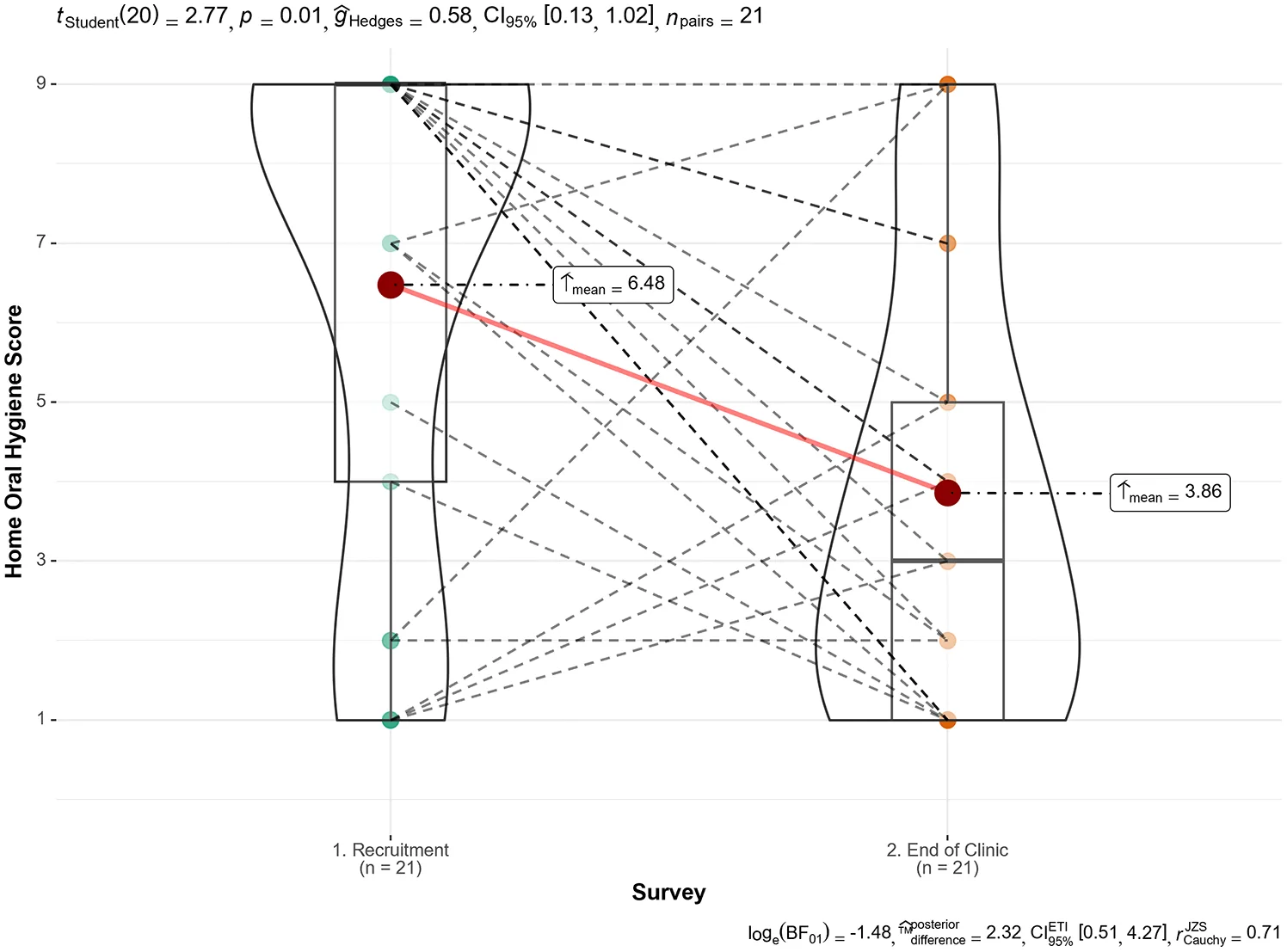
Difference in home oral hygiene score (lower scores correspond to more clinically effective management) across 21 active participants between the recruitment phase and end of clinic phase.

However, this difference did not remain significant when looking at the 13 participants who were followed throughout the study (Fig 5). HOH score did show improvement across the time points, but with only 13 participants, the study was not sufficiently powered to demonstrate a significant effect (F_Fisher_(1.64, 19.63) = 3.28, *p* = .07, ω^2 _p_ = 0.08, 95%CI[0.00, 1.00]).

**Fig 5:**
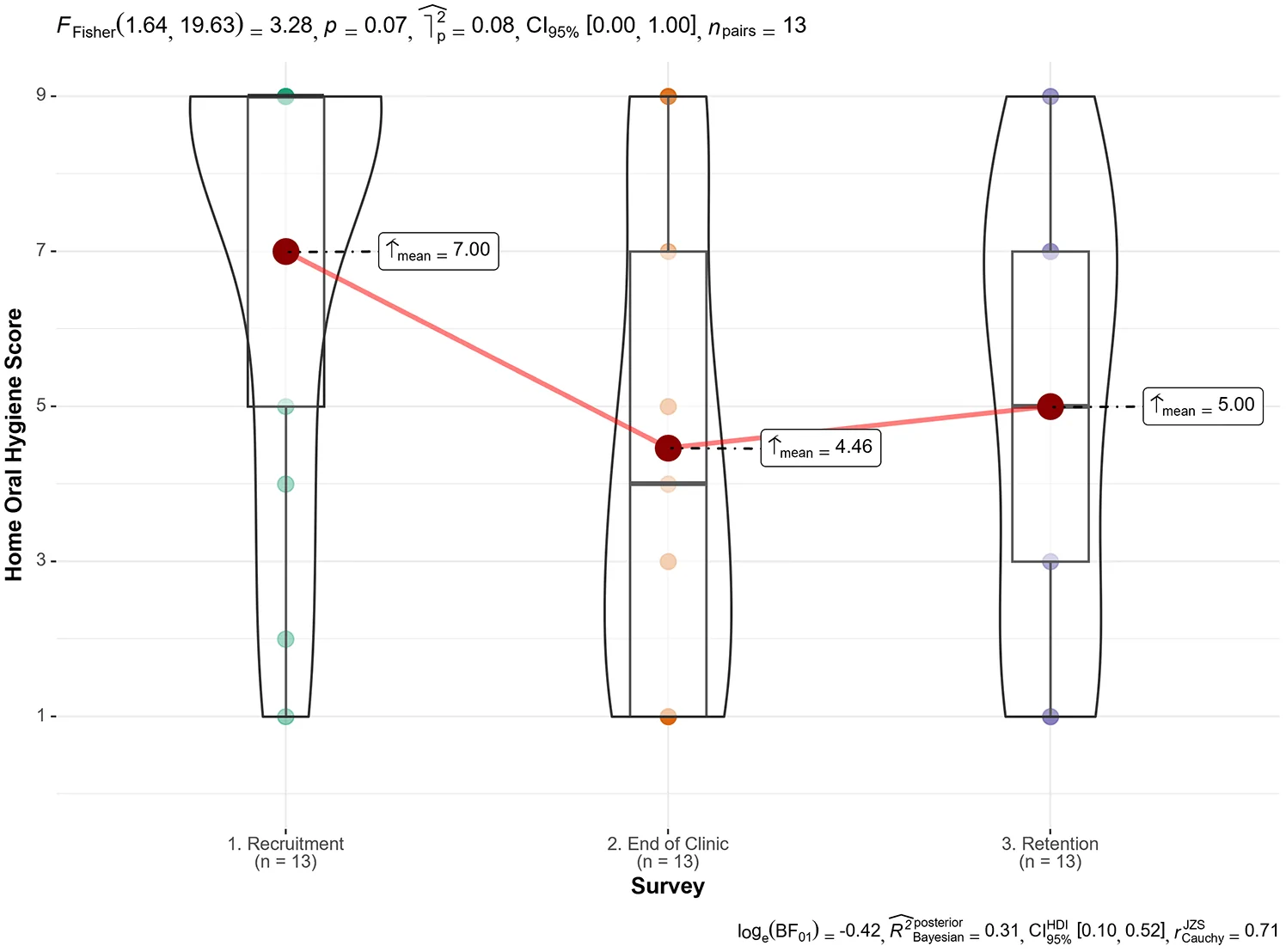
Difference in home oral hygiene score (lower scores correspond to more clinically effective management) across 13 caregivers followed throughout dental clinic intervention.

### Evaluation of clinic

In the End of Clinic survey, 86% of caregivers were satisfied or very satisfied with the consult experience, however 14% (n = 3) reported being very dissatisfied. One of these participants was already maintaining good HOH practice, and upon reviewing clinical notes, it is possible their advice was not clearly personalized for them. Another participant had expressed frustration regarding perceived ineffectiveness and difficulty of brushing, and the third participant had achieved their main goal of reducing halitosis despite reporting dissatisfaction. All participants agreed that the consult discussed their current practice, and the clinician gave them a recommendation.

In the Retention period, 40% of caregivers did not feel they had achieved their goal (one of whom could not remember their goal), and 47% felt their pet’s dental health had not improved following the clinic (one caregiver felt their pet’s health had become worse). 33% of caregivers wanted to be doing something differently to manage their pet’s health, mainly being able to brush the teeth more consistently (n = 4) with one caregiver wanting to try options other than supplements. The majority of caregivers (79%) were using products from the approved list given after the first appointment. The route between goal achievement, happiness with pet’s dental health, and pet’s dental health was not clear. When comparing the Retention responses with the clinical notes, 5 (33%) participants who expressed some unhappiness or desire to be doing better had actually achieved everything they were advised to do in the consult, suggesting that there was a misalignment between the owner’s expectations and clinician’s assessment.

### Thematic analysis on practitioner perspective

The structure, content, and importantly ‘space’ of the 5A framework offers the clinician an approach to contextualised care that is often lacking in normal General Practice. The veterinary consult is often a point of mismatched expectations between caregiver and veterinarian [59], and veterinarians can utilise defensive consult strategies after bad experiences [60]. While both practitioners reported that having a template to follow sometimes felt clunky, the form of the 5A framework itself was seen to have benefits, particularly where follow-up was integrated so the consult remained a conversation with the client.

*“I thought that the follow up would be really helpful in engaging owners because often you give them advice about, oh, your pet could do with a dental treatment or we recommend that you start using this and then you don’t see them again for several months. So actually the telephone check in I felt was quite useful.” – CH*

One aspect of the clinic that surprised the practitioners was the utility of dedicated space, and the transformative nature that had on the consult. As diagnosis of a condition was not a component of the consult, the clinic offered a highly valuable, unique time and space for conversing with the caregiver. This has been observed in ‘new puppy’ consults, which are more preventative, and is rarer in the adult animal space, where caregivers often feel their needs are not met [59].

*“I just had during the dental clinics, I had more time and it was focused on that subject. So it was actually really nice being able to chat to owners” – CH*

Finally, the enforcement to ask a client about their readiness to change, agree on a plan together, and discuss potential challenges ensured that the HOH method chosen from the HOH grading scale was personalised for that patient and client. Combined with the evidence informed grading scale, this allowed the practitioner to pick the most appropriate care option for each patient

“*I know what treatments I’m going to do before they walk in, they need to do preventative oral healthcare so that bit is the bit I normally do is working out where the treatments going to be. Well contextualising it, let’s come up with a plan that works for you.”* – LEW

## Discussion

In this study, we applied a clinician-developed, human behaviour change consult model in a veterinary dental clinic to explore whether it would result in long-term behavioural changes in HOH management. To our knowledge, this is the first instance of the 5A framework being adapted for use in the veterinary context, and one of a limited number of studies exploring how human behavioural change theories can support companion animal welfare. We showed that a Dental Clinic based on the 5As framework creates short-term increases in the frequency of HOH behaviours such as mechanically brushing the teeth (H2), and short-term improvements in overall HOH practice (H3), but we were unable to demonstrate conclusive maintenance of these behaviours in a 6 month retention period, perhaps due to the large drop out rate from the study.

A modified 5As framework could be used in GP veterinary practice by MRCVS and RVN without additional training to elicit caregiver behaviour change to improve preventative healthcare for pets. More research is needed to confirm the effectiveness of this modified model. This study supports the development of future studies to explore the use of human behaviour change in healthcare models in the GP veterinary setting.

### Caregiver HOH knowledge

An unexpected finding was the reversal in HOH effectiveness rankings following the clinic, with 22 participants putting Chews above Mechanical Brushing in the End of Clinic survey in Condorcet Runn Off voting. While we recognise that knowledge alone cannot promote behaviour change, accurate knowledge is still an important cornerstone of any behavioural change [61,62]. Therefore it is important to improve our understanding of this finding.

We have considered possible explanations for this finding. There are generally three possible reasons why a finding might be wrong [54], that it reflects underlying bias in the data, that finding is picking up a confounding effect, and that the finding reflects a lack of sampling power. We discuss the self-selection limitation of the study below, but arguably these caregivers are motivated to engage and attend the clinic, and arguably should be more likely to have a solid knowledge base. Similarly, we address the low power within the study below, but Condorcet Run-Off voting does not require a statistical threshold of significance, instead it is a mathematical representation of a group’s ranked preference [45]. This leaves us with the possibility that the finding is ‘true’ in that it reflects what the participants told us, but it is picking an underlying confounding effect. When reviewing the raw data, the individuals who gave chews a higher rank in the End of Clinic survey were ones who had been advised chews and were consequently using them. Therefore, this finding may represent a recalibration of perceived knowledge to avoid cognitive dissonance and guilt for not brushing. Additionally, it may reflect a misunderstanding in the emphasis of the veterinary advice, that in recommending that chews are the most effective HOH method for this individual patient in a contextualised care approach, the careegiver has extrapolated to assume it is the best for every patient, regardless of their specific contexts. We think this finding might represent an interesting development in recent discussions regarding the tension between ‘gold standard care’ and ‘contextualised care’ [29]. ‘Gold Standard Care’ carries with it an express statement that there is only one correct way to do something, which does not marry well with the aforementioned complex and endlessly unique forms of the caregiver-companion animal bond and patient temperament and severity of periodontal disease. Instead, ‘contextualised care’ aims to integrate the range of appropriate treatments available with recognition of the individual circumstances of the caregiver and patient [63]. In this case, when we asked participants to rank the effectiveness of HOH methods, and thus within context, the caregivers may be fairly assessing Chews as more useful. LEW and CH highlight that they would frequently use the phrase within the clinics, “a chew every day is better than a toothbrush on the shelf”. We think it’s important to clearly state that contextualised care is not about accepting ineffective therapies [64], but if we are arguing that ‘gold standard’ (brushing) is not appropriate in the context of that patient [65], we must accept that chews may represent a valid, contextualised and effective choice which is appropriate within the Spectrum of Care available for each patient. Therefore, caregivers may recognise the efficacy because of their accessibility and ease. This represents an area within the contextualised care that requires a great deal more in-depth qualitative exploration to understand how effective care manifests for caregivers.

### Supporting behaviour change in companion animal caregivers

The caregiver-vet relationship is similar but not directly comparable to the patient-doctor relationship, as the caregiver and decision maker is not the patient themselves. Instead there is a triad of care between veterinarian, caregiver, and animal, this has similarities to paediatric models, which require specialized child orientated and patient orientated communication [66,67]. Paediatric management plans are advised to include the child where possible [68], but how do we do this effectively with pets? The ‘empowerment model’ of the veterinary-caregiver relationship promotes the veterinarian as both a knowledgeable expert, but also a counsellor who supports caregivers to make decisions, but recognises there needs to be critical review in light of the caregiver’s understanding of their specific context [69]. In this study, we found the 5As framework supported caregivers to make short-term changes to HOH behaviours that, per dental management guidance [13], would protect and support animal dental health. Within this, the veterinary clinician must advocate for the animal patient and understand what is appropriate for the patient when the caregiver wants an action which may be a clinical best practice recommendation, but inappropriate for that particular animal. In this study, there were patients who would not tolerate mechanical tooth brushing, and so they were given advice tailored to that patient and caregiver, such as the introduction of chews instead. How veterinarians navigate the challenge of ‘this is best practice overall, but this is best practice for you right now’ is still to be determined. While we were not able to demonstrate a longer-term retention effect, this is a promising approach to providing preventative healthcare in veterinary settings. Individual practices could set up these forms of clinics with a 5As approach to tackle a range of issues. This supports the expert-generated recommendations on how preventative healthcare fits into veterinary practice, including particularly supporting conversations between practitioner and caregiver [70]. Furthermore, these ‘diagnosis-free’ consultations can be supported by veterinary nurses and receptionists in their respective roles, supporting conversations and arranging follow-ups, reflecting the inter-professional practice in veterinary settings [71].

Future research should focus on exploring other consult models, including possibly geriatric care models and reduced-capacity models which reflect similar triads of care [72]. Gathering more data regarding how behaviour change operates in veterinary contexts, similar to work happening in production contexts [26], is also important. Furthermore, robust qualitative research into the less tangible elements of this approach, can help characterise the underlying phenomena driving caregiver decision making.

### Contextualised care

When we were conceptualising this study, we were interested in the 5As Framework because of its specific attention to tailoring advice to individual situations, an important aspect of effective behavioural change in veterinary contexts [18,27]. The study design was approved in February 2024, following review in January 2024. Much of the most recent discussion surrounding contextualised care in veterinary work was prompted in 2021 [29], with influential publications in December 2023 [18] and 2025 [65]. While this study did not explicitly set out to test a Contextualised Care approach, we consider the 5As Framework as having significant overlap with these discussions. The 5As framework encourages exploration of a caregivers’ readiness and barriers to change, which is similar to the communication methods and open questioning recommended to identify the wider context when using the spectrum of care model [44]. Equally, the identification of challenges and co-creation of solutions is very similar to shared decision making, especially in cases where we had multiple appropriate clinical options available. While it is important to remember that the aim of Human Behavioural Change models, like the 5As Framework, and Contextualised Care ethos are not exactly the same, they both recognise the importance of personalised care. Both could benefit from exploring how to support caregivers and decision makers in areas where best-for-you may not be best-overall.

### Limitations

One of the foremost limitations of this work was the low retention rate resulting in a small sample size.

We hoped to recruit 10% of caregivers seen in the recruitment period, and achieved this, however the retention throughout the study phases was disappointing. As part of our institution’s ethical review, we are encouraged to limit the number of times participant personal data is held, which led us to ask caregivers to create their ID label. However, it is highly likely that had we elected to retain participant email addresses, the ability to personalise survey invites and track responses would have resulted in greater retention.

We could have increased our response numbers by increasing our initial sample pool, but this was deemed counterproductive. It was not possible to invite all registered caregivers via email due to GDPR legislation. We elected to recruit at vaccination to support a representative demographic of patients and caregivers. If we invited all caregivers who attended at any time, our sampling would have been skewed towards older and more unwell patients who visit the hospital more frequently. But in recruiting at vaccination we were recruiting for caregivers already participating in preventative care. Anecdotally, the majority of caregivers presenting at the Small Animal General Practice clinic are those who are very motivated to care for their animals, and the vast majority of patients are vaccinated. There is always a challenge of studying disengaged populations.

Overall, approximately 20% (n = 105) of caregivers seen in this period gave consent to be contacted. While there was no means to record if a caregiver refused to be contacted, we assume based on our response numbers that not all caregivers were asked. It is therefore possible that there was an unintended selection criteria in that an animal with poor oral hygiene or a caregiver asking about HOH may have acted as a reminder for the vet or receptionist to ask for consent to be contacted regarding the study. In selecting caregivers visiting for vaccination, we were selecting for caregivers who already demonstrated positive behaviours in preventative healthcare.

## Conclusions

In this study, we demonstrated that a human consult model aimed at promoting health-conscious behaviours, the 5As framework, has application in veterinary settings. Animal caregivers can be supported in veterinary consultations to make positive welfare choices for their animals, and this behaviour can be maintained, at least in the short term. Moreover, this model of consult and approach to preventative healthcare supports contextualised care by engaging caregivers in an empowering model style conversation with the veterinary practitioner, and has the potential for improving animal health and welfare.
