# Supplementary material for: Promoting human behaviour change in a first opinion veterinary clinic to improve home oral hygiene in pets utilising the 5A framework
Source: PLoS One. 2026 Nov 13;21(11):e0362316. doi: 10.1371/journal.pone.0362316 (PMC13766295; doi:10.1371/journal.pone.0362316)
Supplement: Supplementary file 1 [file pone.0362316.s002.docx]

Date : 11.10.2026

Manuscript ID: PONE-D-26-20824R1

Title: Article Type:

Research ArticleFull Title:

Promoting Human Behaviour Change in a First Opinion Veterinary Clinic to improveHome Oral Hygiene in Pets Utilising the 5A Framework

Dear Sir/Mom:

I would like to inform you that this research paper and its objectives were clear and well-defined. The manuscript can now be accepted for publication in the Journal of Plos One after all required revisions have been made by the authors.

Yours sincerely,

Dr. Waddhaah M. Alasbahy
