# Supplementary material for: Promoting human behaviour change in a first opinion veterinary clinic to improve home oral hygiene in pets utilising the 5A framework
Source: PLoS One. 2026 Nov 13;21(11):e0362316. doi: 10.1371/journal.pone.0362316 (PMC13766295; doi:10.1371/journal.pone.0362316)
Supplement: Supplementary file 2 [file pone.0362316.s003.docx]

Dear Dr Sun,

We would like to extend our thanks to the reviewer for their constructive feedback. We have addressed each point, and detailed the response below.

Please note that line numbers refer to approx. location in the clean manuscript.

With kind regards,

| Reviewer | Comment | Response |
| --- | --- | --- |
| Editor | 1.Please ensure that your manuscript meets PLOS ONE's style requirements, including those for file naming. The PLOS ONE style templates can be found at  https://journals.plos.org/plosone/s/file?id=wjVg/PLOSOne_formatting_sample_main_body.pdf and  <https://journals.plos.org/plosone/s/file?id=ba62/PLOSOne_formatting_sample_title_authors_affiliations.pdf> | Title page has been reformatted  Fig files have been resaved as .tif and renamed.  Manuscript reformatted as follows:  Figure changed to Fig throughout  Fig titles inserted after first reference |
|  | 2. Please ensure that you refer to Figure 3 in your text as, if accepted, production will need this reference to link the reader to the figure. | Figure 3 referred to in text in Changes to Behaviour, approx. Line 383 |
|  | 3. Please include a separate caption for each figure in your manuscript. | All figure captions uploaded separately in the portal.  Fig titles/captions inserted into manuscript after first reference. |
|  | 4. If the reviewer comments include a recommendation to cite specific previously published works, please review and evaluate these publications to determine whether they are relevant and should be cited. There is no requirement to cite these works unless the editor has indicated otherwise. | The reviewer gave no recommendations for specific works |
| 1 | 4. Is the manuscript presented in an intelligible fashion and written in standard English?  PLOS ONE does not copyedit accepted manuscripts, so the language in submitted articles must be clear, correct, and unambiguous. Any typographical or grammatical errors should be corrected at revision, so please note any specific errors here.  Reviewer #1: No | With thanks to your detailed comments below, and additionally our minor changes throughout, we hope this has been addressed. |
|  | Reviewer #1: Thank you for an interesting paper, and for seeking to publish despite the small sample size.  I think there are a couple of areas where the paper could be strengthened. | We thank the reviewer for their constructive comments (and also noting the sample size – we agree!) |
|  | 1. Please give a stronger rationale for the use of the 5As model of behaviour change, when, as stated, this is its first use in veterinary healthcare. Why was the COM-B model dismissed, when it has been used in several previous studies, and was the use of motivational interviewing considered? | We have added some materials throughout explaining our rationale, particularly:  Introduction (Approx Line 98)  Much of the current work around supporting animal welfare through human behavioural change has been limited in applying human behavioural change theory (27). A recent review in production animals (26) found that most human behavioural change studies focus around Theory of Planned Behaviour Change, and most studies focused on behavioural intention, rather than the behaviour itself. These studies were also not related to clinical care behaviours, e.g. behaviours that we would expect to see as a consequence of clinical consultation.  And approx. Line 113  We were interested in whether the 5As framework could support animal caregivers to promote HOH behaviours for two key reasons. While many other behavioural change models have been utilised in animal welfare contexts, such as Theory of Planned Behavioural Change, Behavioural Change Wheel, the Health Action Process Approach, and COM-B models, their use is centred around designing an educational intervention, and occasionally testing it (26). Clinical treatments do not always benefit from conceptualised as educational interventions, and this impacts how behavioural change theories can be utilised within them (36). The 5As framework was specifically designed and utilised for use in consults, and has been found effective in this setting. It required minimal training for veterinary clinicians to use, and could be quickly integrated into clinical practice. Additionally, as the 5As framework had been successfully utilised in paediatric settings, we were interested in whether it was flexible enough to adapt to the veterinary triad of care between practitioner, animal caregiver, and animal.  And Approx Line 129  The 5As framework has clearly set out actions for the clinician (Ask, Advise, Assess, Assist, Arrange) which can be easily incorporated into routine General Practice utilising vet and nurse core communication skills, unlike other human behavioural change approaches such as motivational interviewing and the COM-B model which require personnel training and advanced, new, or additional communication skills. To our knowledge, this is the first research study exploring the 5As framework in the veterinary context |
|  | 2. Please rephrase the term ‘5As consult model’. The 5As is a specific behaviour change approach, not a model for all consultations (e.g., the Calgary Cambridge model, the Four Habits model), so it should not be given this title. I would strongly recommend a change in the title and throughout the text. | We have removed any reference to the 5As Consult Model, and for clarity referred to the 5As Framework throughout, consistent with literature e.g. Shieh et al 2024 |
|  | 3. I am sceptical that the approach used was one that incorporated contextualised care (CC). | With thanks to you for this comment which prompted a great deal of discussion among the authors.  Overall, we feel that Contextualised Care does not mean that caregivers are not made aware of best practice, but that they are given support to make changes that are valuable and actionable for them.  To address this comment, we have taken a number of steps, which we have detailed in the below comments.  In the introduction, we reference Contextualised Care to illustrate its embedding within veterinary practice. We reference ‘Spectrum of Care’ as part of dental care approx. Line 73 and highlight that all patient-client interactions are unique at approx. Line 93  Within the results, we have reflected on the quote that you highlighted to demonstrate that the clinician was considering the clinic format as a whole:  (Approx Lines 449).  Finally, the enforcement to ask a client about their readiness to change, agree on a plan together, and discuss potential challenges ensured that the HOH method chosen from the HOH grading scale was personalised for that patient and client. Combined with the evidence informed grading scale, this allowed the practitioner to pick the most appropriate care option for each patient  “I know what treatments I'm going to do before they walk in, they need to do preventative oral healthcare so that bit is the bit I normally do is working out where the treatments going to be. Well contextualising it, let's come up with a plan that works for you.” – LEW  And within the discussion, we have added a new section on ‘Contextualised Care’, approx. Line 552  **Contextualised Care**  When we were conceptualising this study, we were interested in the 5As Framework because of its specific attention to tailoring advice to individual situations, an important aspect of effective behavioural change in veterinary contexts (18,27). The study design was approved in February 2024, following review in January 2024. Much of the most recent discussion surrounding contextualised care in veterinary work was prompted in 2021 (29), with influential publications in December 2023 (18) and 2025 (64). While this study did not explicitly set out to test a Contextualised Care approach, we consider the 5As Framework as having significant overlap with these discussions. The 5As framework encourages exploration of a clients’ readiness and barriers to change, which is similar to the communication methods and open questioning recommended to identify the wider context when using the spectrum of care model (72). Equally, the identification of challenges and co-creation of solutions is very similar to shared decision making, especially in cases where we had multiple appropriate clinical options available. While it is important to remember that the aim of Human Behavioural Change models, like the 5As Framework, and Contextualised Care ethos are not exactly the same, they both recognise the importance of personalised care. Both could benefit from exploring how to support caregivers and decision makers in areas where best-for-you may not be best-overall. |
|  | To use CC effectively, the veterinary professional would need to find out more about the contexts involved and offer a range of options. In this study, it was clear that brushing with a toothbrush was the ‘gold standard’ and was the option that was heavily promoted. Indeed, the quote from one of the veterinary professionals “The clinical plan that I need is already set out is the same for every patient. It's just about the contextualising it” shows a basic misunderstanding of the fundamental approach to CC. | Within the methodology, we have highlighted further how the ‘assist’ step used contextualised care  Approx Line 180  Within the ‘Assist’ step, a contextualised care approach (18) was utilised to evaluate each caregiver-patient dyad’s scope for change. We highlight to readers that we provided our recommendations with a stated optimum care plan, e.g. mechanically brushing teeth with a paste, following clinical recommendations (13), but that for each caregiver-patient dyad there was a specific care plan set out which may have recommended a less-optimal approach per clinical recommendations, but was contextualised to that dyad’s requirements. |
|  | Using a CC approach would involve 1. finding out about the animal’s temperament, the client’s circumstances in terms of time available, finances and physical capability, their views on preventative healthcare, other family members involved and what they think/provide, the involvement of the veterinary healthcare team in terms of what is available at the practice etc., 2. offering the range of options and helping the client to make the best decision for them and their pet in their particular circumstances and 3. supporting that decision with regular check-ins. It is not clear whether this approach was utilised – maybe it was, but if so, it should be described in the paper. If it was not, then please clearly identify that a CC approach was not used but might be useful in the future in view of the findings of the study. | We feel we did incorporate these considerations within the consults, and have elaborated further  approx. Line 200:  appropriate for that individual patient, taking into account the patient’s behavioural needs, personality, the caregiver’s finances, capacity, and physical capabilities to recommend advice that was achievable for the caregiver-patient dyad, and of clinical benefit. This meant, for example, that some caregivers were not recommended mechanical toothbrushing if the patient would not tolerate the procedure. During the Arrange step, at follow-up, the personalised plan was re-assessed to explore whether the individual care plan was working, and changes could be made if not, with further follow-up possible.  And Approx Line 225  Each practitioner was free to make their own assessment on acceptable methods for example ensuring that any HOH method chose would not worsen any mouth pain, or not recommending brushing if the patient’s temperament could put the client at risk of being bitten.  At approx. Line 285  The HOH Grading scale demonstrates the clinical effectiveness of each daily HOH method. While the HOH grading scale was primarily used as an outcome measure to determine whether clinically-relevant improvement in HOH practice was observed, it was also , used as a guide by the clinicians and clients to pick the best HOH for each individual patient and client with shared decision making (44)  Within the discussion, we have clarified how we view Contextualised Care in the context of the knowledge change findings.  Approx Line 494  Additionally, it may reflect a misunderstanding in the emphasis of the veterinary advice, that in recommending that chews are the most effective HOH method for this individual patient in a contextualised care approach, the client has extrapolated to assume it is the best for every patient, regardless of their specific contexts.  Approx Line. 528  Within this, the veterinary clinician must advocate for the animal patient and understand what is appropriate for the patient when the caregiver wants an action which may be a clinical best practice recommendation, but inappropriate for that particular animal. In this study, there were patients who would not tolerate mechanical tooth brushing, and so they were given advice tailored to that patient and caregiver, such as the introduction of chews instead. How veterinarians navigate the challenge of ‘this is best practice overall, but this is best practice for you right now’ is still to be determined. |
|  | 4. Although the positioning of one of the authors (JM) is clearly explained, the other authors are merely listed in terms of their current roles. It would be good to include more about their positioning regarding research philosophies. | Position statement has been updated as follows,  Approx Line 159  JRDM was the lead on study design and analysis, and is not a veterinary professional, but has worked in animal and childcare settings for a number of years. She describes herself as principally a methodologist working in veterinary education and animal welfare, with a socio-constructivist epistemological position. LEW is general practice veterinary surgeon with an academic interest and teaching roles in Veterinary Communication client experience, and contextualised care. She is developing her research perspective and is largely post-positivist, reflecting her clinical veterinary training. CH is a registered veterinary nurse with a Veterinary Technician Specialty in Dentistry and a background in clinical veterinary nursing and veterinary dentistry education, FM is a registered veterinary nurse with an oncology speciality and a lecturer in veterinary education with a background in psychology and communication, and AC is a veterinary receptionist. FM, CH and AC did not contribute to the data analysis, and so epistemological positions are not relevant. KH is a veterinarian and researcher in veterinary education, with a socio-constructivist epistemological position. |
|  | Syntax, grammar etc.: Line 39: ‘are’ should be ‘is’ as you are talking about a population, but that makes the sentence a bit clunky; you could reword as ‘Companion animals, as an animal population, are heavily reliant on their caregivers’ | With thanks for the suggestion, rephrased as follows:  Companion animals, as an animal population, are heavily reliant on their caregivers to make informed decisions regarding their health and wellbeing (1). |
|  | Line 54: ‘and affect a range of companion animals from dogs (14), cats (15), and rabbits (16)’ should be ‘and affects a range of companion animals from dogs (14) to cats (15) and rabbits (16)’. | Rephrased as follows:  and affect a range of companion animals from dogs (14) to cats (15) and rabbits (16). |
|  | Line 58: ‘where the bacteria causes inflammation’ should be ‘where the bacteria cause inflammation’ | Rephrased as follows:  where the bacteria cause inflammation of the gingiva ( |
|  | Line 175: ‘The Initial Consult involved an appointment to the Dental Clinic’ should be ‘The Initial Consult involved an appointment with (or at) the Dental Clinic’ | Rephrased as follows:  The Initial Consult involved an appointment with the Dental Clinic |
|  | Lines 180-186: The paragraph ‘with the caregiver being asked which Home Oral Hygiene Methods were being used daily, advised on personalised advise with examples tailored to the patient and lifestyle, assess whether the caregiver was willing to start or adapt their Home Oral Hygiene approach, assisting the caregiver on agreeing a plan with a demonstration, discussion the potential challenges with solutions, and giving the caregiver supplementary materials highlighting information relevant to the caregivers personal circumstances, and finally a follow-up call was booked for 4-8 weeks to arrange a further check’ is used to illustrate the 5As model, but reads rather clumsily due to the change of tense. It would be worth rewriting this paragraph to align tenses and make it flow better, and ‘personalised advise’ should be ‘personalised advice’. | Rephrased as follows:  The 5As were incorporated into the consultation as follows. The caregiver was first **asked** which Home Oral Hygiene Methods were being used daily. The caregiver was given personalised **advice,** with examples tailored to the patient and lifestyle. The clinician made an **assessment** as to whether the caregiver was willing to start or adapt their Home Oral Hygiene approach. The clinician then **assisted** the caregiver to agree on a patient and client centred plan, with a demonstration, discussing the potential challenges with solutions, and giving the caregiver supplementary materials highlighting information relevant to the caregivers personal circumstances. Finally a follow-up call was booked for 4-8 weeks to **arrange** a further check. |
|  | Line 215: ‘And all three surveys’ would be better as ‘All three surveys’ | Rephrased as:  All three surveys |
|  | Line 259: ‘All statistical analysis were performed’ should be ‘All statistical analyses were performed’ | Rephrased as:  All statistical analyses were performed in |
|  | Line 344: A personal one here – I hate the term ‘had gotten’. I know you used it in the survey questions, but please could you replace it with ‘had got’ or ‘had become’ in the text here? | Rephrased as:  one caregiver felt their pet’s health had become worse |
