# Supplementary material for: Promoting human behaviour change in a first opinion veterinary clinic to improve home oral hygiene in pets utilising the 5A framework
Source: PLoS One. 2026 Nov 13;21(11):e0362316. doi: 10.1371/journal.pone.0362316 (PMC13766295; doi:10.1371/journal.pone.0362316)
Supplement: Peer review history

# Peer review: Decision Letter 0

**DOI:** 10.1371/journal.pone.0362316.r001

**Authors:** Xin Sun

1 Jul 2026

Dear Dr. MacKay,

Thank you for submitting your manuscript to PLOS ONE. After careful consideration, we feel that it has merit but does not fully meet PLOS ONE’s publication criteria as it currently stands. Therefore, we invite you to submit a revised version of the manuscript that addresses the points raised during the review process.

Please submit your revised manuscript by Aug 15 2026 11:59PM. If you will need more time than this to complete your revisions, please reply to this message or contact the journal office at plosone@plos.org. When you're ready to submit your revision, log on to https://www.editorialmanager.com/pone/ and select the 'Submissions Needing Revision' folder to locate your manuscript file.

- A letter that responds to each point raised by the academic editor and reviewer(s). You should upload this letter as a separate file labeled 'Response to Reviewers'.
- A marked-up copy of your manuscript that highlights changes made to the original version. You should upload this as a separate file labeled 'Revised Manuscript with Track Changes'.
- An unmarked version of your revised paper without tracked changes. You should upload this as a separate file labeled 'Manuscript'.

If you would like to make changes to your financial disclosure, please include your updated statement in your cover letter. Guidelines for resubmitting your figure files are available below the reviewer comments at the end of this letter.

If applicable, we recommend that you deposit your laboratory protocols in protocols.io to enhance the reproducibility of your results. Protocols.io assigns your protocol its own identifier (DOI) so that it can be cited independently in the future. For instructions see: https://journals.plos.org/plosone/s/submission-guidelines#loc-laboratory-protocols. Additionally, PLOS ONE offers an option for publishing peer-reviewed Lab Protocol articles, which describe protocols hosted on protocols.io. Read more information on sharing protocols at https://plos.org/protocols?utm_medium=editorial-email&utm_source=authorletters&utm_campaign=protocols.

As the corresponding author, your ORCID iD is verified in the submission system and will appear in the published article. PLOS supports the use of ORCID, and we encourage all coauthors to register for an ORCID iD and use it as well. Please encourage your coauthors to verify their ORCID iD within the submission system before final acceptance, as unverified ORCID iDs will not appear in the published article. *Only*  the individual author can complete the verification step; PLOS staff *cannot*  verify ORCID iDs on behalf of authors.

We look forward to receiving your revised manuscript.

Kind regards,

Xin Sun, PhD

Staff Editor

PLOS One

Journal Requirements:

When submitting your revision, we need you to address these additional requirements.

1.Please ensure that your manuscript meets PLOS ONE's style requirements, including those for file naming. The PLOS ONE style templates can be found at

https://journals.plos.org/plosone/s/file?id=wjVg/PLOSOne_formatting_sample_main_body.pdf and

https://journals.plos.org/plosone/s/file?id=ba62/PLOSOne_formatting_sample_title_authors_affiliations.pdf

2. Please ensure that you refer to Figure 3 in your text as, if accepted, production will need this reference to link the reader to the figure.

3. Please include a separate caption for each figure in your manuscript.

4. If the reviewer comments include a recommendation to cite specific previously published works, please review and evaluate these publications to determine whether they are relevant and should be cited. There is no requirement to cite these works unless the editor has indicated otherwise.

Additional Editor Comments:

Your work has been evaluated by one expert in the field. They raised several scientific concerns on rationale and approach of the work. Please read carefully and address them one by one in your revision.

[Note: HTML markup is below. Please do not edit.]

Reviewers' comments:

Reviewer's Responses to Questions

**Comments to the Author**

1. Is the manuscript technically sound, and do the data support the conclusions?

Reviewer #1: Yes

**********

2. Has the statistical analysis been performed appropriately and rigorously?

Reviewer #1: Yes

**********

3. Have the authors made all data underlying the findings in their manuscript fully available?

The PLOS Data policy

Reviewer #1: Yes

**********

4. Is the manuscript presented in an intelligible fashion and written in standard English?

Reviewer #1: No

**********

Reviewer #1: Thank you for an interesting paper, and for seeking to publish despite the small sample size.

I think there are a couple of areas where the paper could be strengthened.

1. Please give a stronger rationale for the use of the 5As model of behaviour change, when, as stated, this is its first use in veterinary healthcare. Why was the COM-B model dismissed, when it has been used in several previous studies, and was the use of motivational interviewing considered?

2. Please rephrase the term ‘5As consult model’. The 5As is a specific behaviour change approach, not a model for all consultations (e.g., the Calgary Cambridge model, the Four Habits model), so it should not be given this title. I would strongly recommend a change in the title and throughout the text.

3. I am sceptical that the approach used was one that incorporated contextualised care (CC). To use CC effectively, the veterinary professional would need to find out more about the contexts involved and offer a range of options. In this study, it was clear that brushing with a toothbrush was the ‘gold standard’ and was the option that was heavily promoted. Indeed, the quote from one of the veterinary professionals “The clinical plan that I need is already set out is the same for every patient. It's just about the contextualising it” shows a basic misunderstanding of the fundamental approach to CC. Using a CC approach would involve 1. finding out about the animal’s temperament, the client’s circumstances in terms of time available, finances and physical capability, their views on preventative healthcare, other family members involved and what they think/provide, the involvement of the veterinary healthcare team in terms of what is available at the practice etc., 2. offering the range of options and helping the client to make the best decision for them and their pet in their particular circumstances and 3. supporting that decision with regular check-ins. It is not clear whether this approach was utilised – maybe it was, but if so, it should be described in the paper. If it was not, then please clearly identify that a CC approach was not used but might be useful in the future in view of the findings of the study.

4. Although the positioning of one of the authors (JM) is clearly explained, the other authors are merely listed in terms of their current roles. It would be good to include more about their positioning regarding research philosophies.

Syntax, grammar etc.:

Line 39: ‘are’ should be ‘is’ as you are talking about a population, but that makes the sentence a bit clunky; you could reword as ‘Companion animals, as an animal population, are heavily reliant on their caregivers’

Line 54: ‘and affect a range of companion animals from dogs (14), cats (15), and rabbits (16)’ should be ‘and affects a range of companion animals from dogs (14) to cats (15) and rabbits (16)’.

Line 58: ‘where the bacteria causes inflammation’ should be ‘where the bacteria cause inflammation’

Line 175: ‘The Initial Consult involved an appointment to the Dental Clinic’ should be ‘The Initial Consult involved an appointment with (or at) the Dental Clinic’

Lines 180-186: The paragraph ‘with the caregiver being asked which Home Oral Hygiene Methods were being used daily, advised on personalised advise with examples tailored to the patient and lifestyle, assess whether the caregiver was willing to start or adapt their Home Oral Hygiene approach, assisting the caregiver on agreeing a plan with a demonstration, discussion the potential challenges with solutions, and giving the caregiver supplementary materials highlighting information relevant to the caregivers personal circumstances, and finally a follow-up call was booked for 4-8 weeks to arrange a further check’ is used to illustrate the 5As model, but reads rather clumsily due to the change of tense. It would be worth rewriting this paragraph to align tenses and make it flow better, and ‘personalised advise’ should be ‘personalised advice’.

Line 215: ‘And all three surveys’ would be better as ‘All three surveys’

Line 259: ‘All statistical analysis were performed’ should be ‘All statistical analyses were performed’

Line 344: A personal one here – I hate the term ‘had gotten’. I know you used it in the survey questions, but please could you replace it with ‘had got’ or ‘had become’ in the text here?

**********

what does this mean?). If published, this will include your full peer review and any attached files.

If you choose “no”, your identity will remain anonymous but your review may still be made public.

**Do you want your identity to be public for this peer review?**  For information about this choice, including consent withdrawal, please see our Privacy Policy

Reviewer #1: No

**********

[NOTE: If reviewer comments were submitted as an attachment file, they will be attached to this email and accessible via the submission site. Please log into your account, locate the manuscript record, and check for the action link "View Attachments". If this link does not appear, there are no attachment files.]

To ensure your figures meet our technical requirements, please review our figure guidelines: https://journals.plos.org/plosone/s/figures

You may also use PLOS’s free figure tool, NAAS, to help you prepare publication quality figures: https://journals.plos.org/plosone/s/figures#loc-tools-for-figure-preparation.

NAAS will assess whether your figures meet our technical requirements by comparing each figure against our figure specifications.

# Author comment: Author response to Decision Letter 1

**DOI:** 10.1371/journal.pone.0362316.r002

9 Aug 2026

Please see attached Response to Reviewers for detailed comments. We thank the reviewer for their constructive comments, and hope we have addressed them satisfactorily.

Dear Dr Sun,

We would like to extend our thanks to the reviewer for their constructive feedback. We have addressed each point, and detailed the response below.

Please note that line numbers refer to approx. location in the clean manuscript.

With kind regards,

Editor Response

Please ensure that your manuscript meets PLOS ONE's style requirements, including those for file naming. The PLOS ONE style templates can be found at

https://journals.plos.org/plosone/s/file?id=wjVg/PLOSOne_formatting_sample_main_body.pdf and

https://journals.plos.org/plosone/s/file?id=ba62/PLOSOne_formatting_sample_title_authors_affiliations.pdf

RESPONSE: Title page has been reformatted

Fig files have been resaved as .tif and renamed.

Manuscript reformatted as follows:

Figure changed to Fig throughout

Fig titles inserted after first reference

2. Please ensure that you refer to Figure 3 in your text as, if accepted, production will need this reference to link the reader to the figure.

RESPONSE: Figure 3 referred to in text in Changes to Behaviour, approx. Line 383

3. Please include a separate caption for each figure in your manuscript.

RESPONSE: All figure captions uploaded separately in the portal.

Fig titles/captions inserted into manuscript after first reference.

4. If the reviewer comments include a recommendation to cite specific previously published works, please review and evaluate these publications to determine whether they are relevant and should be cited. There is no requirement to cite these works unless the editor has indicated otherwise.

RESPONSE: The reviewer gave no recommendations for specific works

Reviewer Responses

4. Is the manuscript presented in an intelligible fashion and written in standard English?

PLOS ONE does not copyedit accepted manuscripts, so the language in submitted articles must be clear, correct, and unambiguous. Any typographical or grammatical errors should be corrected at revision, so please note any specific errors here.

Reviewer #1: No

RESPONSE:

With thanks to your detailed comments below, and additionally our minor changes throughout, we hope this has been addressed.

Reviewer #1: Thank you for an interesting paper, and for seeking to publish despite the small sample size.

I think there are a couple of areas where the paper could be strengthened.

RESPONSE: We thank the reviewer for their constructive comments (and also noting the sample size – we agree!)

1. Please give a stronger rationale for the use of the 5As model of behaviour change, when, as stated, this is its first use in veterinary healthcare. Why was the COM-B model dismissed, when it has been used in several previous studies, and was the use of motivational interviewing considered?

RESPONSE:

We have added some materials throughout explaining our rationale, particularly:

Introduction (Approx Line 98)

Much of the current work around supporting animal welfare through human behavioural change has been limited in applying human behavioural change theory (27). A recent review in production animals (26) found that most human behavioural change studies focus around Theory of Planned Behaviour Change, and most studies focused on behavioural intention, rather than the behaviour itself. These studies were also not related to clinical care behaviours, e.g. behaviours that we would expect to see as a consequence of clinical consultation.

And approx. Line 113

We were interested in whether the 5As framework could support animal caregivers to promote HOH behaviours for two key reasons. While many other behavioural change models have been utilised in animal welfare contexts, such as Theory of Planned Behavioural Change, Behavioural Change Wheel, the Health Action Process Approach, and COM-B models, their use is centred around designing an educational intervention, and occasionally testing it (26). Clinical treatments do not always benefit from conceptualised as educational interventions, and this impacts how behavioural change theories can be utilised within them (36). The 5As framework was specifically designed and utilised for use in consults, and has been found effective in this setting. It required minimal training for veterinary clinicians to use, and could be quickly integrated into clinical practice. Additionally, as the 5As framework had been successfully utilised in paediatric settings, we were interested in whether it was flexible enough to adapt to the veterinary triad of care between practitioner, animal caregiver, and animal.

And Approx Line 129

The 5As framework has clearly set out actions for the clinician (Ask, Advise, Assess, Assist, Arrange) which can be easily incorporated into routine General Practice utilising vet and nurse core communication skills, unlike other human behavioural change approaches such as motivational interviewing and the COM-B model which require personnel training and advanced, new, or additional communication skills. To our knowledge, this is the first research study exploring the 5As framework in the veterinary context

2. Please rephrase the term ‘5As consult model’. The 5As is a specific behaviour change approach, not a model for all consultations (e.g., the Calgary Cambridge model, the Four Habits model), so it should not be given this title. I would strongly recommend a change in the title and throughout the text.

RESPONSE: We have removed any reference to the 5As Consult Model, and for clarity referred to the 5As Framework throughout, consistent with literature e.g. Shieh et al 2024

3. I am sceptical that the approach used was one that incorporated contextualised care (CC). With thanks to you for this comment which prompted a great deal of discussion among the authors.

RESPONSE: Overall, we feel that Contextualised Care does not mean that caregivers are not made aware of best practice, but that they are given support to make changes that are valuable and actionable for them.

To address this comment, we have taken a number of steps, which we have detailed in the below comments.

In the introduction, we reference Contextualised Care to illustrate its embedding within veterinary practice. We reference ‘Spectrum of Care’ as part of dental care approx. Line 73 and highlight that all patient-client interactions are unique at approx. Line 93

Within the results, we have reflected on the quote that you highlighted to demonstrate that the clinician was considering the clinic format as a whole:

(Approx Lines 449).

Finally, the enforcement to ask a client about their readiness to change, agree on a plan together, and discuss potential challenges ensured that the HOH method chosen from the HOH grading scale was personalised for that patient and client. Combined with the evidence informed grading scale, this allowed the practitioner to pick the most appropriate care option for each patient

“I know what treatments I'm going to do before they walk in, they need to do preventative oral healthcare so that bit is the bit I normally do is working out where the treatments going to be. Well contextualising it, let's come up with a plan that works for you.” – LEW

And within the discussion, we have added a new section on ‘Contextualised Care’, approx. Line 552

Contextualised Care

When we were conceptualising this study, we were interested in the 5As Framework because of its specific attention to tailoring advice to individual situations, an important aspect of effective behavioural change in veterinary contexts (18,27). The study design was approved in February 2024, following review in January 2024. Much of the most recent discussion surrounding contextualised care in veterinary work was prompted in 2021 (29), with influential publications in December 2023 (18) and 2025 (64). While this study did not explicitly set out to test a Contextualised Care approach, we consider the 5As Framework as having significant overlap with these discussions. The 5As framework encourages exploration of a clients’ readiness and barriers to change, which is similar to the communication methods and open questioning recommended to identify the wider context when using the spectrum of care model (72). Equally, the identification of challenges and co-creation of solutions is very similar to shared decision making, especially in cases where we had multiple appropriate clinical options available. While it is important to remember that the aim of Human Behavioural Change models, like the 5As Framework, and Contextualised Care ethos are not exactly the same, they both recognise the importance of personalised care. Both could benefit from exploring how to support caregivers and decision makers in areas where best-for-you may not be best-overall.

Reviewer:

To use CC effectively, the veterinary professional would need to find out more about the contexts involved and offer a range of options. In this study, it was clear that brushing with a toothbrush was the ‘gold standard’ and was the option that was heavily promoted. Indeed, the quote from one of the veterinary professionals “The clinical plan that I need is already set out is the same for every patient. It's just about the contextualising it” shows a basic misunderstanding of the fundamental approach to CC.

RESPONSE:

Within the methodology, we have highlighted further how the ‘assist’ step used contextualised care

Approx Line 180

Within the ‘Assist’ step, a contextualised care approach (18) was utilised to evaluate each caregiver-patient dyad’s scope for change. We highlight to readers that we provided our recommendations with a stated optimum care plan, e.g. mechanically brushing teeth with a paste, following clinical recommendations (13), but that for each caregiver-patient dyad there was a specific care plan set out which may have recommended a less-optimal approach per clinical recommendations, but was contextualised to that dyad’s requirements.

REVIEWER:

Using a CC approach would involve 1. finding out about the animal’s temperament, the client’s circumstances in terms of time available, finances and physical capability, their views on preventative healthcare, other family members involved and what they think/provide, the involvement of the veterinary healthcare team in terms of what is available at the practice etc., 2. offering the range of options and helping the client to make the best decision for them and their pet in their particular circumstances and 3. supporting that decision with regular check-ins. It is not clear whether this approach was utilised – maybe it was, but if so, it should be described in the paper. If it was not, then please clearly identify that a CC approach was not used but might be useful in the future in view of the findings of the study.

RESPONSE: We feel we did incorporate these considerations within the consults, and have elaborated further

approx. Line 200:

appropriate for that individual patient, taking into account the patient’s behavioural needs, personality, the caregiver’s finances, capacity, and physical capabilities to recommend advice that was achievable for the caregiver-patient dyad, and of clinical benefit. This meant, for example, that some caregivers were not recommended mechanical toothbrushing if the patient would not tolerate the procedure. During the Arrange step, at follow-up, the personalised plan was re-assessed to explore whether the individual care plan was working, and changes could be made if not, with further follow-up possible.

And Approx Line 225

Each practitioner was free to make their own assessment on acceptable methods for example ensuring that any HOH method chose would not worsen any mouth pain, or not recommending brushing if the patient’s temperament could put the client at risk of being bitten.

At approx. Line 285

The HOH Grading scale demonstrates the clinical effectiveness of each daily HOH method. While the HOH grading scale was primarily used as an outcome measure to determine whether clinically-relevant improvement in HOH practice was observed, it was also , used as a guide by the clinicians and clients to pick the best HOH for each individual patient and client with shared decision making (44)

Within the discussion, we have clarified how we view Contextualised Care in the context of the knowledge change findings.

Approx Line 494

Additionally, it may reflect a misunderstanding in the emphasis of the veterinary advice, that in recommending that chews are the most effective HOH method for this individual patient in a contextualised care approach, the client has extrapolated to assume it is the best for every patient, regardless of their specific contexts.

Approx Line. 528

Within this, the veterinary clinician must advocate for the animal patient and understand what is appropriate for the patient when the caregiver wants an action which may be a clinical best practice recommendation, but inappropriate for that particular animal. In this study, there were patients who would not tolerate mechanical tooth brushing, and so they were given advice tailored to that patient and caregiver, such as the introduction of chews instead. How veterinarians navigate the challenge of ‘this is best practice overall, but this is best practice for you right now’ is still to be determined.

REVIEWER: 4. Although the positioning of one of the authors (JM) is clearly explained, the other authors are merely listed in terms of their current roles. It would be good to include more about their positioning regarding research philosophies.

RESPONSE: Position statement has been updated as follows,

Approx Line 159

JRDM was the lead on study design and analysis, and is not a veterinary professional, but has worked in animal and childcare settings for a number of years. She describes herself as principally a methodologist working in veterinary education and animal welfare, with a socio-constructivist epistemological position. LEW is general practice veterinary surgeon with an academic interest and teaching roles in Veterinary Communication client experience, and contextualised care. She is developing her research perspective and is largely post-positivist, reflecting her clinical veterinary training. CH is a registered veterinary nurse with a Veterinary Technician Specialty in Dentistry and a background in clinical veterinary nursing and veterinary dentistry education, FM is a registered veterinary nurse with an oncology speciality and a lecturer in veterinary education with a background in psychology and communication, and AC is a veterinary receptionist. FM, CH and AC did not contribute to the data analysis, and so epistemological positions are not relevant. KH is a veterinarian and researcher in veterinary education, with a socio-constructivist epistemological position.

Syntax, grammar etc.:

Line 39: ‘are’ should be ‘is’ as you are talking about a population, but that makes the sentence a bit clunky; you could reword as ‘Companion animals, as an animal population, are heavily reliant on their caregivers’

RESPONSE: With thanks for the suggestion, rephrased as follows:

Companion animals, as an animal population, are heavily reliant on their caregivers to make informed decisions regarding their health and wellbeing (1).

Line 54: ‘and affect a range of companion animals from dogs (14), cats (15), and rabbits (16)’ should be ‘and affects a range of companion animals from dogs (14) to cats (15) and rabbits (16)’.

RESPONSE: Rephrased as follows:

and affect a range of companion animals from dogs (14) to cats (15) and rabbits (16).

Line 58: ‘where the bacteria causes inflammation’ should be ‘where the bacteria cause inflammation’

RESPONSE: Rephrased as follows:

where the bacteria cause inflammation of the gingiva (

Line 175: ‘The Initial Consult involved an appointment to the Dental Clinic’ should be ‘The Initial Consult involved an appointment with (or at) the Dental Clinic’

RESPONSE: Rephrased as follows:

The Initial Consult involved an appointment with the Dental Clinic

Lines 180-186: The paragraph ‘with the caregiver being asked which Home Oral Hygiene Methods were being used daily, advised on personalised advise with examples tailored to the patient and lifestyle, assess whether the caregiver was willing to start or adapt their Home Oral Hygiene approach, assisting the caregiver on agreeing a plan with a demonstration, discussion the potential challenges with solutions, and giving the caregiver supplementary materials highlighting information relevant to the caregivers personal circumstances, and finally a follow-up call was booked for 4-8 weeks to arrange a further check’ is used to illustrate the 5As model, but reads rather clumsily due to the change of tense. It would be worth rewriting this paragraph to align tenses and make it flow better, and ‘personalised advise’ should be ‘personalised advice’.

RESPONSE: Rephrased as follows:

The 5As were incorporated into the consultation as follows. The caregiver was first asked which Home Oral Hygiene Methods were being used daily. The caregiver was given personalised advice, with examples tailored to the patient and lifestyle. The clinician made an assessment as to whether the caregiver was willing to start or adapt their Home Oral Hygiene approach. The clinician then assisted the caregiver to agree on a patient and client centred plan, with a demonstration, discussing the potential challenges with solutions, and giving the caregiver supplementary materials highlighting information relevant to the caregivers personal circumstances. Finally a follow-up call was booked for 4-8 weeks to arrange a further check.

Line 215: ‘And all three surveys’ would be better as ‘All three surveys’

RESPONSE: Rephrased as:

All three surveys

Line 259: ‘All statistical analysis were performed’ should be ‘All statistical analyses were performed’

RESPONSE: Rephrased as:

All statistical analyses were performed in

Line 344: A personal one here – I hate the term ‘had gotten’. I know you used it in the survey questions, but please could you replace it with ‘had got’ or ‘had become’ in the text here?

________________________________________

RESPONSE: Rephrased as:

one caregiver felt their pet’s health had become worse

Attachment Submitted filename: Response to Reviewers.docx

# Peer review: Decision Letter 1

**DOI:** 10.1371/journal.pone.0362316.r003

**Authors:** Nayan Ranjan Singha

22 Oct 2026

Promoting Human Behaviour Change in a First Opinion Veterinary Clinic to improve Home Oral Hygiene in Pets   Utilising the 5A Framework

PONE-D-26-20824R1

Dear Dr. Jill MacKay,

We’re pleased to inform you that your manuscript has been judged scientifically suitable for publication and will be formally accepted for publication once it meets all outstanding technical requirements.

Within one week, you’ll receive an e-mail detailing the required amendments. When these have been addressed, you’ll receive a formal acceptance letter and your manuscript will be scheduled for publication.

An invoice will be generated when your article is formally accepted. Please note, if your institution has a publishing partnership with PLOS and your article meets the relevant criteria, all or part of your publication costs will be covered. Please make sure your user information is up-to-date by logging into Editorial Manager at Editorial Manager® and clicking the ‘Update My Information' link at the top of the page. For questions related to billing, please contact billing support.

If your institution or institutions have a press office, please notify them about your upcoming paper to help maximize its impact. If they’ll be preparing press materials, please inform our press team as soon as possible -- no later than 48 hours after receiving the formal acceptance. Your manuscript will remain under strict press embargo until 2 pm Eastern Time on the date of publication. For more information, please contact onepress@plos.org.

Kind regards,

Nayan Ranjan Singha, Ph.D.

Academic Editor

PLOS One

Additional Editor Comments (optional):

Reviewers' comments:

Reviewer's Responses to Questions

**Comments to the Author**

Reviewer #1: All comments have been addressed

Reviewer #2: All comments have been addressed

**********

2. Is the manuscript technically sound, and do the data support the conclusions?

Reviewer #1: Yes

Reviewer #2: Yes

**********

3. Has the statistical analysis been performed appropriately and rigorously?

Reviewer #1: Yes

Reviewer #2: Yes

**********

4. Have the authors made all data underlying the findings in their manuscript fully available?

The PLOS Data policy

Reviewer #1: Yes

Reviewer #2: Yes

**********

5. Is the manuscript presented in an intelligible fashion and written in standard English?

Reviewer #1: Yes

Reviewer #2: No

**********

Reviewer #1: Thank you for your comprehensive response. I am convinced by your argument that you tried to incorporate a contextualised care approach into the consultations, and I feel that the revisions to the manuscript have improved it with respect to the rationale for the use of the 5As framework and the use of contextualised care.

Reviewer #2: (No Response)

**********

what does this mean?). If published, this will include your full peer review and any attached files.

If you choose “no”, your identity will remain anonymous but your review may still be made public.

**Do you want your identity to be public for this peer review?**  For information about this choice, including consent withdrawal, please see our Privacy Policy

Reviewer #1: **Yes:**  Carol Gray

Reviewer #2: No

**********

Attachment Submitted filename: Plos One 002.docx

# Editor report: Acceptance letter

**DOI:** 10.1371/journal.pone.0362316.r004

**Authors:** Nayan Ranjan Singha

PONE-D-26-20824R1

PLOS One

Dear Dr. MacKay,

I'm pleased to inform you that your manuscript has been deemed suitable for publication in PLOS One. Congratulations! Your manuscript is now being handed over to our production team.

At this stage, our production department will prepare your paper for publication. This includes ensuring the following:

* All references, tables, and figures are properly cited

* All relevant supporting information is included in the manuscript submission,

* There are no issues that prevent the paper from being properly typeset

You will receive further instructions from the production team, including instructions on how to review your proof when it is ready. Please keep in mind that we are working through a large volume of accepted articles, so please give us a few days to review your paper and let you know the next and final steps.

Lastly, if your institution or institutions have a press office, please let them know about your upcoming paper now to help maximize its impact. If they'll be preparing press materials, please inform our press team within the next 48 hours. Your manuscript will remain under strict press embargo until 2 pm Eastern Time on the date of publication. For more information, please contact onepress@plos.org.

You will receive an invoice from PLOS for your publication fee after your manuscript has reached the completed accept phase. If you receive an email requesting payment before acceptance or for any other service, this may be a phishing scheme. Learn how to identify phishing emails and protect your accounts at https://explore.plos.org/phishing.

If we can help with anything else, please email us at customercare@plos.org.

Thank you for submitting your work to PLOS One and supporting open access.

Kind regards,

PLOS One Editorial Office Staff

on behalf of

Dr. PLOS Manuscript Reassignment

Staff Editor

PLOS One
